# Effect of brushing simulation on the wear behavior of repaired CAD-CAM restorations

**DOI:** 10.1016/j.identj.2024.02.012

**Published:** 2024-03-08

**Authors:** Pablo Machado Soares, Amanda Maria de Oliveira Dal Piva, Gabriel Kalil Rocha Pereira, Luiz Felipe Valandro, Marilia Pivetta Rippe, Albert J. Feilzer, Cornelis Johannes Kleverlaan, João Paulo Mendes Tribst

**Affiliations:** a Division of Prosthodontics-Biomaterials, Center for Development of Advanced Materials, Federal University of Santa Maria (UFSM), Santa Maria, Brazil; b Department of Dental Materials Science, Academic Centre for Dentistry Amsterdam (ACTA), Universiteit van Amsterdam and Vrije Universiteit, Amsterdam, North Holland, The Netherlands; c Department of Reconstructive Oral Care, Academic Centre for Dentistry Amsterdam (ACTA), Universiteit van Amsterdam and Vrije Universiteit, Amsterdam, North Holland, The Netherlands

**Keywords:** Dental restoration repair, Surface roughness, Surface Loss, Toothbrush wear

## Abstract

**Objective:**

The aim of the present study was to evaluate the influence of multidirectional brushing on the surface roughness, morphology, and bonding interface of resin-repaired CAD-CAM ceramic and composite restorations. Materials and methods: Twelve (*N* = 12) blocks (4 mm × 4 mm × 2 mm for parallel axis; 5 mm × 4 mm × 2 mm for perpendicular axis) of lithium disilicate glass-ceramic (IPS e.max CAD, Ivoclar AG) and CAD-CAM resin composite (Tetric CAD, Ivoclar AG) were obtained and repaired with direct resin composite (Clearfil AP-X, Kuraray). An abrasive slurry was prepared and the brushing was performed according to each restorative material and axis of brushing (*n* = 6; perpendicular to repair interface and parallel to repair interface) during 3,650 cycles (240 strokes per minute) to simulate 3 years of brushing. The surface roughness (Ra) and the profile variation for each material (restoration and direct repair resin composite) were measured at the baseline condition and after brushing, and the mean roughness and presence of steps at the repair interface were evaluated through factorial analysis of Variance (ANOVA). Scanning Electron Microscopy (SEM) images were taken to evaluate the surface topography of the repaired materials after brushing.

**Results:**

The mean roughness of the repaired CAD-CAM restorations was affected by the brushing (*P* < .05), mainly when evaluating the repair material and the interface (*P* < .05), while the restorative CAD-CAM materials presented more stable values. The profile evaluation showed higher steps at the interface when repairing lithium disilicate than for CAD-CAM resin composite.

**Conclusion:**

Repaired CAD-CAM restorations were susceptible to wear after brushing simulation. The surface roughness of the direct resin composite was the most affected leading to step development at the interface, particularly in the repaired lithium disilicate samples. Cinical maintenance recalls and polishing protocols must be considered to enhance the longevity of such restorations.

## Introduction

The computer-aided design and Computer-aided manufacturing (CAD-CAM) system has been widely used for oral rehabilitation. In this sense, lithium disilicate glass-ceramic is one of the most popular restorative materials, considering its versatility which combines satisfactory mechanical strength and excellent aesthetic provided by its crystalline and vitreous content.^1^ These properties make this material suitable for both posterior and anterior restorations,^2^, ^3^, ^4^ where high aesthetics are demanded.

Despite the effectiveness and excellent survival rates of monolithic lithium disilicate crows (83.5% in 10 years),^3^ technical complications such as fractures have been reported.^2^ If a ceramic restoration fails, clinicians have to decide whether to replace the crown or opt for repair procedures using resin composite. It is crucial to use repair protocols whenever possible. Compared to the restoration replacement, direct repair is the most cost-effective option, since it minimizes the necessity for extensive tooth preparation, and can be accomplished in a single session.^5^^,^^6^ Additionally, previous studies reported that repaired-ceramic restorations are effective in terms of bond strength to resin-based materials when etched with fluorotic acid or self-etch ceramic primers.^7^, ^8^, ^9^ However, the stability and integrity of the repaired interface is still a theme of concern, since the resin composite material is more susceptible to wear stimulus when compared to ceramic materials.^10^^,^^11^

Resin-based materials have also been developed for the CAD-CAM system, in order to decrease the wear of the antagonist's tooth and provide indirect restorations that mimic the teeth's properties as elastic modulus, consequently reducing the occurrence of fractures commonly seen in brittle materials.^12^, ^13^, ^14^ Besides, even if failures can still occur, the repair of composite restorations is easier due to the similar microstructure between resin-based materials through their chemical compatibility and well-described surface treatments and bonding agents.^15^^,^^16^ However, resin composite restorations may not perform as well if their surface wear and roughness are increased. Previous studies corroborate that surface alterations can affect their colour properties and mechanical behavior,^17^^,^^18^ as same as the bonding interface when repair procedures were performed.

Additionally, the surface roughness of restorative materials can be affected by toothbrushing.^19^ It has been reported that both ceramic and composite materials showed different morphologic characteristics and roughness after abrasion by brushing.^20^^,^^21^ Also, the combined use of dental brushes and abrasives, such as dentifrices, can accelerate the wear effect, besides the increase of porosity, water absorption, and colour change.^20^^,^^22^, ^23^, ^24^ In this sense, simulated mechanical brushing has been reported as a suitable method to evaluate the effect of normal hygiene procedures on the surface characteristics of dental materials.^25^ However, to the author's knowledge, there is still no study reporting the effect of such procedures on the wear of repaired ceramic and composite restorations, especially when considering the bonding interface of the repair.

Considering the aforementioned factors, the aim of the present study was to evaluate the influence of multidirectional brushing on the surface roughness, morphology and bonding interface of resin-repaired CAD-CAM ceramic and composite restorations. The hypotheses were that the brushing would affect (1) the surface roughness and morphology, (2) and the bonding interface of the repaired restorations.

## Materials and methods

### Specimen preparation

Ceramic and composite (*N* = 12) CAD-CAM blocks (IPS e.max CAD, Ivoclar AG; Tetric CAD, Ivoclar AG) were attached to metallic holders and sectioned into smaller slices in a precision cutting machine (Isomet 1000, Buehler) under constant water cooling. The blocks were then grounded and polished with #400, #600, and #1200 Silica Carbide papers (SiC) until achieve their final dimensions (4 mm × 4 mm × 2 mm for parallel axis; 5 mm × 4 mm × 2 mm for perpendicular axis) and absence of superficial defects. All specimens were measured with a digital calliper to assure the same dimensions and positioning of the interface during the brushing test. The lithium disilicate blocks were crystalized in a specific furnace (10 min at 840°C, 7 min vacuum), according to the manufacturer guidelines.

### Surface treatments and repair with resin composite

**Fig. 1:**
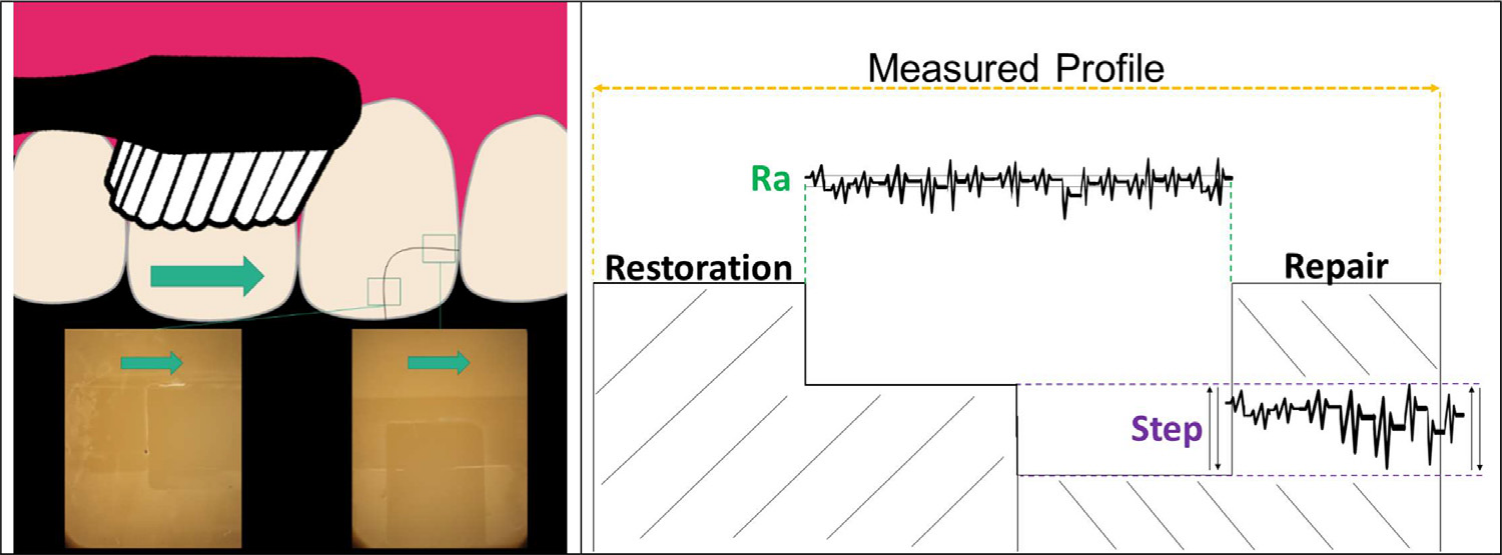
Illustration of the 2 axis of brushing (perpendicular and parallel to the repaired interface and surface aspect after the brushing protocol (left). Description of the measured roughness parameters (right). The yellow lines indicate the measured area for the specimen's profile. The green lines indicate the mean roughness (Ra) measurement. The purple lines indicate the evaluation of the wear discrepancy between the restorative and repair materials after the brushing protocol, depicted by the difference in the profile mensuration for the restorative and repair material.

The blocks were randomly divided into 4 experimental groups (*n* = 6) according to 2 factors under study: restorative material (lithium disilicate or CAD-CAM resin composite), and axis of brushing movement (parallel to the bonding interface of the repair and perpendicular to the bonding interface of the repair) (Figure 1). All specimens were cleaned in an ultrasonic bath for 10 min with ethanol 90%, and then the proper surface treatments were performed according to the material to be repaired, as follows:

*For lithium disilicate glass-ceramic:* A self-etching ceramic primer (Monobond Etch & Prime, Ivoclar AG) was applied actively on the bonding surface of the specimens with a microbrush for 15 s and then left to react for 40 s. After that, the product was removed with air-water spray for 30 s and air-dried for another 30 s.

*For CAD-CAM resin composite:* The specimens were air-abraded with alumina particles (50µm grain size) for 10 s at 2 bar of pressure and air-dried for the removal of any debris. A universal adhesive system (Adhese Universal, Ivoclar AG) was applied for 20 s on the composite bonding surface, gently air-dried to remove the excess product, and then light-cured (Elipar FreeLight 2, 3M) for 20 s.

Following the surface treatments, the specimens were affixed to rectangular mounts (10 × 8 × 6 mm) using adhesive tape. Initially, resin composite (Clearfil AP-X, Kuraray) was applied to the bonding surface of the CAD-CAM materials, mimicking a repair interface. Subsequently, the composite was filled throughout the device to ensure complete coverage. Eventually, the resulting rectangular blocks were carefully removed from the mounts, and uniformity was achieved by polishing with #600 and #1200 SiC until all specimens reached a consistent height of 2 mm.

### Surface roughness analysis

Before the brushing test, the surface roughness of all specimens was measured in a contact profilometer (SJ-400, Mitutoyo), as a baseline condition. Six measurements were taken for each specimen at a speed of 0.2 mm/s and range of 2 mm,^21^ being 2 measurements for the restoration (lithium disilicate or CAD-CAM resin composite), 2 for the repair resin composite, and another 2 for the interface in each axis. The analysis was performed according to ISO 4287:1997, and Ra (arithmetical mean of roughness in μm) of only the brushed area was measured at the baseline, and after 3 years of brushing to compare the roughness in the restoration, the repair material and the interface.

Finally, the profile variation of the surface was also evaluated for each group in order to evaluate the possible development of a step at the interface after the 3-year brushing simulation (Figure 1). The profile was measured for the restorative material and then for the adjacent repair resin composite by using the remote mode of the profilometer. The obtained data were then moved to a specific software (SJ-tools, Mitutoyo), which generate and exported the profiles in graphics. The difference between the profile of both materials was calculated to determine the step value (between repair and restoration) for each repaired restoration after brushing.

### Abrasive slurry preparation and brushing simulation

An abrasive slurry was prepared by mixing 25g of silica reference abrasive (9 µm particle size - Sident, Grace, Worms, Germany) with 40 ml of distillate water until a homogenous mixture was obtained.

A multistation (6 stations) brushing machine (PMT; Tamson Instruments) was used to simulate the brushing procedures for each group per time. Metallic devices were positioned over the specimens to restrict the brushing area (2.5 × 8 mm), while the covered area was used as a reference for the evaluation of the wear pattern. Six soft brushes (M41, Lactona) were fixed at the arms of the machine, with all the bristles in the same direction of the brushing area, parallel to each other.^21^ The abrasive slurry was applied over each station until covering all the specimens of the group. The specimens were firstly brushed for 3,650 cycles (240 strokes per minute, with 2.45 N being applied by the toothbrushes) according to each group (parallel and perpendicular to the interface), which was reported as a simulation of 1 year of toothbrushing.^26^ In order to simulate 3 years of brushing, the mentioned procedure was carried out 3 times. At the end of each test, the abrasive solution was discarded, the specimens were cleaned with distillate water and the surface roughness was measured as mentioned.

### Scanning electron microscopy

After the brushing tests and surface roughness analysis, 1 representative specimen of each group was selected for a surface topography analysis through scanning electron microscopy (SEM, Evo LS15, Carl Zeiss, Gottingen, Germany), to evaluate the pattern or wear for the CAD-CAM materials and for the repair resin composite, as same as to evaluate the repair interface, at magnifications of 65× and 500×.

### Statistical analysis

The normality and homoscedasticity of the obtained data were evaluated by the Shapiro-Wilk and Levene tests, respectively.

As the data assumed a normal distribution, a factorial analysis of Variance (3-Way ANOVA) was carried out to evaluate the mean roughness (Ra) in the brushed area for each axis of brushing according to each factor under study: brushing (aging); local (restorative or repair material); and restoration (Lithium disilicate or CAD-CAM resin composite). Two-way ANOVA was performed to evaluate the profile roughness for each material after the brushing simulation and the discrepancy (step) of wear between the restorative and the repair material in the brushed area, considering local and restoration (lithium disilicate or CAD-CAM resin composite).

## Results

The ANOVA results are depicted in Table 1. The mean roughness (Ra) of the brushed area for the restoration, repair material and interface for each axis of brushing is depicted in Table 2. Considering the parallel axis, Three-Way ANOVA showed that the location (*P* < .05; F = 9.79) and brushing (*P* < .05; F = 28.80) factors affected the mean surface in the brushed area, while the restoration factor (*P* = .447; F = 0.59) and their interaction did not (*P* = .645; F = 0.44). For the perpendicular axis, the location (*P* = .444; F = 0.82), restoration (*P* = .355; F = 0.87) and the interaction of factors (*P* = .188; F = 1.73 ) did not affect the mean Ra, while the brushing factor (*P* < .05; F = 26.20) influenced the results. For both axes, it is clear that the 3-year brushing simulation increased the mean roughness, mainly when the measurements included the repair material (repair itself and repaired interface). On the other hand, the roughness of the CAD-CAM materials was more stable after the brushing.

**Table 1:** Three-way ANOVA for mean surface roughness (Ra) and 2-way ANOVA for profile evaluation for each axis.

Three-way ANOVA for Ra evaluation					
Factors	Sun of squares	DF	Mean square	F	*P*
**Parallel**					
Brushing	1.86	1	1.86	28.80	<.0001
Local	1.27	2	0.63	9.79	<.001
Material	0.04	1	0.04	0.59	.045
Interaction	0.06	2	0.03	0.44	.645
Error	3.10	48	0.06		
Total	7.67	59			
**Perpendicular**					
Brushing	2.03	1	2.03	26.20	<.0001
Local	0.13	2	0.06	0.82	.445
Material	0.07	1	0.07	0.87	.356
Interaction	0.27	2	0.13	1.73	.188
Error	3.71	48	0.08		
Total	7.19	59			

**Two-way ANOVA for the profile evaluation**					

**Parallel**					
Local	129.82	1	129.82	26.68	<.0001
Restorative material	0.17	1	0.17	0.03	.854
Interaction	79.75	1	79.75	16.39	<.0001
Error	175.15	36	4.87		
Total	384.88	39			
**Perpendicular**					
Local	91.02	1	91.02	5.19	.028
Restorative material	14.76	1	14.76	0.84	.364
Interaction	126.52	1	126.52	7.22	.010
Error	631.18	36	17.53		
Total	863.49	39

**Table 2:** Three-way ANOVA for mean surface roughness (Ra) at the ceramic, repair composite and interface according to the axis of brushing.

Groups	Brushing axis	Ra substrate	Ra repair composite	Ra interface
**LD**	**Parallel**	0.126 (0.025)^C^	0.246 (0.191)^BC^	0.118 (0.019)^C^
**Tetric**		0.256 (0.210)^BC^	0.216 (0.200)^BC^	0.128 (0.037)^C^
**LD brushed**		0.126 (0.026)^C^	0.694 (0.141)^AB^	0.686 (0.154)^AB^
**Tetric brushed**		0.120 (0.046)^C^	0.826(0.607)^A^	0.752 (0.486)^AB^
**LD**	**Perpendicular**	0.116 (0.015)^B^	0.138 (0.044)^B^	0.156 (0.076)^AB^
**Tetric**		0.354 (0.258)^AB^	0.154 (0.066)^B^	0.122 (0.039)^B^
**LD brushed**		0.294 (0.230)^AB^	0.590 (0.417)^AB^	0.648 (0.364)^AB^
**Tetric brushed**		0.800 (0.495)^A^	0.672 (0.477)^AB^	0.242 (0.118)^AB^

The capital letters indicate statistical differences for each brushing axis according to the factors under study: brushing (aging); local (restorative or repair material); and restoration (Lithium disilicate or CAD-CAM resin composite) depicted by 3-way ANOVA.

**Fig. 2:**
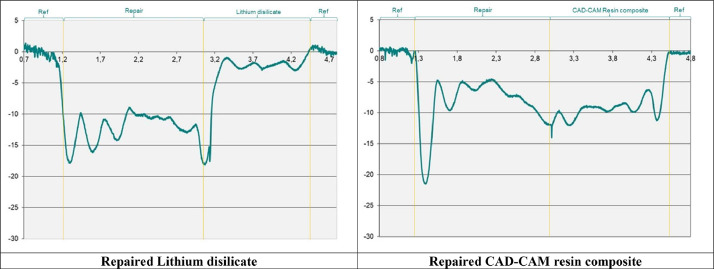
Profile graphics after 3 years of brushing simulation for repaired lithium disilicate glass-ceramic (left) and repaired CAD-CAM resin composite (right).

The profile results after brushing are depicted in Table 3. The discrepancy (Step) of profiles in the interface between the restoration and the repair material was also evaluated before and after brushing (Table 3, Figure 2) For both axis, 2-way ANOVA showed that only the restoration factor did not affect the specimen's profile (parallel: *P* = .854, F = 0.03; perpendicular: *P* = .364, F = 0.84), while the location (parallel: *P* < .05, F = 26.68; perpendicular: *P* = .028, F = 5.19) and the interaction (parallel: *P* < .05, F = 16.39; perpendicular: *P* = .010, F = 7.22) influenced the results. The profile values showed a higher difference between the lithium disilicate and repair composite when compared to the differences between the CAD-CAM resin composite and its repair material, corroborating for a higher step at the interface when repairing dental ceramics.

**Table 3:** Two-way ANOVA for profile evaluation between restorative and repair materials, according to the axis of brushing.

**Groups**	**Brushing axis**	**Profile**	**Step difference**
**LD**	**Parallel**	−2.508 (0.730)^A^	6.43
**Repair LD**		−8.935 (1.490)^C^	
**Tetric**		−5.461 (3.050)^B^	0.78
**Repair Tetric**		−6.240 (3.030)^B^	
**LD**	**Perpendicular**	−2.483 (3.730)^A^	6.57
**Repair LD**		−9.057 (5.480)^B^	
**Tetric**		−4.825 (2.820)^AB^	0.54
**Repair Tetric**		−4.285 (2.390)^AB^	

The capital letters indicate statistical differences for each brushing axis according to the factors local (restorative or repair material); and restoration (Lithium disilicate or CAD-CAM resin composite) depicted by 2-way ANOVA.

Step difference between the substrate and repair profiles.

**Fig. 3:**
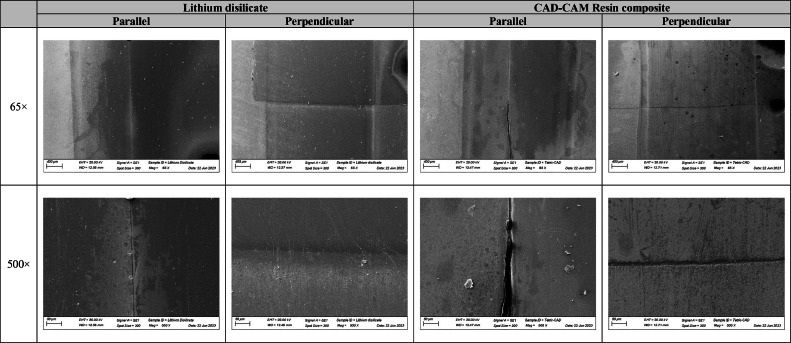
SEM images of the CAD-CAM materials (lithium disilicate and CAD-CAM resin composite) depicting the repaired interface parallel and perpendicular to the brushing simulation.

The SEM images are depicted in Figure 3. The brushed area is evident for both axis and materials, as it modified the surface aspect of the specimens, mainly when considering only composite materials. Also, more scratches can be seen for the repaired CAD-CAM resin composite when compared to the lithium disilicate.

## Discussion

The brushing simulation demonstrated an impact on the surface of the repaired restorative materials, consequently affecting the bonding interface of the repair. Additionally, the mean roughness and profile pattern of the evaluated materials increased for both brushing axes (parallel and perpendicular). Therefore, the adopted hypothesis was accepted.

Among the evaluated materials, the direct repair resin composite showed the highest values of mean roughness (Ra) followed by the CAD-CAM resin composite, while the lithium disilicate glass-ceramic depicted lower and stable values of roughness when comparing the baseline and brushed conditions. Direct resin-based materials are known as excellent and versatile options for restorative purposes,^14^^,^^27^^,^^28^ due to their good mechanical properties and polishing potential, which make these materials suitable for aesthetic restorations.^27^ However, they also present a higher wear susceptibility in the presence of mechanical and abrasive stimulus when compared to CAD-CAM resin composites and dental ceramics,^11^ which was corroborated by the present study (Table 2). This may be explained by the microstructure of the evaluated nanohybrid direct resin composite, that includes nano and micro sized fillers in the resin matrix^27^ as well as the layering technique that can incorporate defects and voids, and due to the unconverted monomers. Despite their compositional similarities, the polymerization method of CAD-CAM resin composites differs significantly and boasts greater efficiency compared to that of direct resin composites. This distinction involves the application of elevated temperatures and specific pressure conditions, which collectively enhance the ultimate outcomes in relation to mechanical properties like hardness and resistance to abrasion.^29^

The CAD-CAM ceramic materials present excellent mechanical behaviour and aesthetic pleasant, provided by their microstructure that contains a glass-matrix and also crystal arrangement in the case of lithium disilicate.^1^ In this sense, ceramic materials are harder and more resistant to abrasion than resin composites. When considering the lithium disilicate glass-ceramic, the mean roughness was lowered after brushing (Table 2), suggesting a polishing effect caused by the brushing protocol. This may also explain the stable wear behaviour of ceramic after the brushing simulation, since the mean roughness was less affected when compared to the resin-based materials when considering the parallel axis. Previous studies showed that ceramic materials are also less susceptible to bacterial adhesion and pigmentation than resin composite since they present more polishing stability.^30^^,^^31^ Thus, in can be expected that the lithium disilicate would show a higher longevity in terms of discoloration and biological complications as secondary caries. However, recalls and maintenance seems to be still essential for repolishing and follow-up when the crown is repaired by direct resin composite. Previous studies showed that polishing protocols may be effective to revert pigmentation and reduce the surface roughness of resin composite and dental ceramics.^32^^,^^33^

Regarding to the profiles for each restorative material, lithium disilicate demonstrated a more homogenous and protruding profile than resin composite materials, mainly when considering the parallel axis of brushing (Table 3, Figure 2). Besides, the difference in profile pattern at the interface was more evident between lithium disilicate and repair composite than between the CAD-CAM resin composite and its repair (Table 3). These findings are in accordance with previous studies,^11^^,^^34^ corroborating that resin-based materials are more affected by the mechanical brushing and use of abrasives. For repaired CAD-CAM resin composite, the profile pattern was deeper but also homogenous between the restorative and repair materials (Figure 2), being the step at the interface lower, probably due to the closer wear behaviour and higher similarity of polymeric materials when compared to the ceramic.^11^ As a result, a less conspicuous interface and reduced complications, such as biofilm accumulation, are anticipated at the interface of resin-based restorations. In this context, occasional polishing procedures should suffice to augment the durability of these materials. Nevertheless, when mending lithium disilicate with direct resin composites, the microstructural disparities between the 2 materials are likely to lead to more pronounced deterioration over time. As consequence, the step at the interface between the repair and the ceramic was higher, which would probably be detected by the patient itself in time. Thus, the need for more frequent maintenance recalls for polishing procedures or even the replacement of the repair may be also higher.

As limitations of the present study, it can be mentioned that in the oral environment, there are other important factors that affect the repair interface and wear behaviour of restorative materials, such as pH, temperature oscillations, and diet. However, the evaluation of the mechanical effects of toothbrushing is still necessary, mainly when different materials are involved in the restoration. In this sense, in vitro studies are important to evaluate such effects in a controlled scenario, and future studies should also evaluate the effects of different factors, such as abrasives, other resin composites for repair and CAD-CAM materials.

## Conclusion

Both repaired CAD-CAM ceramic and resin composite restorations were significantly affected by brushing simulation. The surface roughness of the direct resin composite was the most affected local, generating steps at the repair interface, mainly when considering repaired lithium disilicate. Thus, maintenance recalls and polishing protocols must be considered to enhance the longevity of such restorations.

## CRediT authorship contribution statement

**Pablo Machado Soares:** Conceptualization, Methodology, Software, Validation, Formal analysis, Investigation, Data curation, Writing – original draft, Writing – review & editing, Visualization. **Amanda Maria de Oliveira Dal Piva:** Investigation, Writing – review & editing, Visualization, Supervision, Project administration. **Gabriel Kalil Rocha Pereira:** Investigation, Resources, Writing – review & editing, Visualization, Supervision, Project administration. **Luiz Felipe Valandro:** Resources, Writing – review & editing, Visualization, Project administration. **Marilia Pivetta Rippe:** Resources, Writing – review & editing, Visualization, Project administration, Funding acquisition. **Albert J. Feilzer:** Investigation, Writing – review & editing, Visualization, Supervision, Project administration. **Cornelis Johannes Kleverlaan:** Conceptualization, Resources, Investigation, Writing – review & editing, Visualization, Supervision, Project administration, Funding acquisition. **João Paulo Mendes Tribst:** Conceptualization, Methodology, Investigation, Writing – review & editing, Visualization, Supervision, Project administration.

## Conflict of interest

The authors declare that they have no known competing financial interests or personal relationships that could have appeared to influence the work reported in this paper.

## References

[1] Zarone F, Di Mauro MI, Ausiello P, Ruggiero G, Sorrentino R (2019). Current status on lithium disilicate and zirconia: a narrative review. BMC Oral Health.

[2] Pieger S, Salman A, Bidra A. (2014). Clinical outcomes of lithium disilicate single crowns and partial fixed dental prostheses: a systematic review. J Prosthet Dent.

[3] Rauch A, Reich S, Dalchau L, Schierz O. (2018). Clinical survival of chair-side generated monolithic lithium disilicate crowns: 10-year results. Clin Oral Investig.

[4] Aziz A, El-Mowafy O, Paredes S. (2020). Clinical outcomes of lithium disilicate glass-ceramic crowns fabricated with CAD/CAM technology: a systematic review. Dent Med Probl.

[5] Federation FWD (2017). FDI policy statement on minimal intervention dentistry (MID) for managing dental caries: adopted by the general assembly: September 2016, Poznan, Poland. Int Dent J.

[6] Sanal FA, Kilinc H. (2020). Evaluating ceramic repair materials in terms of bond strength and color stability. Int J Prosthodont.

[7] Scherer MM, Prochnow C, Venturini AB, Pereira GKR, Burgo TAL, Rippe MP (2018). Fatigue failure load of an adhesively-cemented lithium disilicate glass-ceramic: conventional ceramic etching vs etch & prime one-step primer. Dent Mater.

[8] Ueda N, Takagaki T, Nikaido T, Takahashi R, Ikeda M, Tagami J. (2021). The effect of different ceramic surface treatments on the repair bond strength of resin composite to lithium disilicate ceramic. Dent Mater J.

[9] Simasetha S, Klaisiri A, Sriamporn T, Sappayatosok K, Thamrongananskul N. (2022). Surface treatment effect on shear bond strength between lithium disilicate glass-ceramic and resin cement. Eur J Dent.

[10] Zhang H, Lv P, Du W, Jiang T. (2020). Comparison of fracture load and surface wear of microhybrid composite and ceramic occlusal veneers. J Prosthodont.

[11] Ximinis E, Dionysopoulos D, Papadopoulos C, Tournavitis A, Konstantinidis A, Naka O. (2023). Effect of tooth brushing simulation on the surface properties of various resin-matrix computer-aided design/computer-aided manufacturing ceramics. J Esthet Restor Dent.

[12] Ilie N, Hickel R. (2011). Resin composite restorative materials. Aust Dent J.

[13] Schlichting LH, Maia HP, Baratieri LN, Magne P. (2011). Novel-design ultra-thin CAD/CAM composite resin and ceramic occlusal veneers for the treatment of severe dental erosion. J Prosthet Dent.

[14] Sripetchdanond J, Leevailoj C. (2014). Wear of human enamel opposing monolithic zirconia, glass ceramic, and composite resin: an in vitro study. J Prosthet Dent.

[15] Zaghloul H, Elkassas DW, Haridy MF. (2014). Effect of incorporation of silane in the bonding agent on the repair potential of machinable esthetic blocks. Eur J Dent.

[16] Arpa C, Ceballos L, Fuentes MV, Perdigão J. (2019). Repair bond strength and nanoleakage of artificially aged CAD-CAM composite resin. J Prosthet Dent.

[17] Lohbauer U, Müller FA, Petschelt A. (2008). Influence of surface roughness on mechanical strength of resin composite versus glass ceramic materials. Dent Mater.

[18] Tavangar M, Bagheri R, Kwon TY, Mese A, Manton DJ. (2018). Influence of beverages and surface roughness on the color change of resin composites. J Investig Clin Dent.

[19] Dal Piva AMO, Bottino MA, Anami LC, Werner A, Kleverlaan CJ, Giudice RL (2021). Toothbrushing wear resistance of stained CAD/CAM ceramics. Coatings.

[20] Ardu S, Braut V, Uhac I, Benbachir N, Feilzer AJ, Krejci I. (2009). Influence of mechanical and chemical degradation on surface gloss of resin composite materials. Am J Dent.

[21] Tribst JPM, Dal Piva AMO, Werner A, Silva LTS, Anami LC, Bottino MA (2021). Effect of surface treatment and glaze application on shade characterized resin-modified ceramic after toothbrushing. J Prosthet Dent.

[22] Zanin FR, Garcia LFR, Casemiro LA (2008). Pires-de-Souza FCP. Effect of artificial accelerated aging on color stability and surface roughness of indirect composites. Eur J Prosthodont Restor Dent.

[23] da Costa J, Adams-Belusko A, Riley K (2010). The effect of various dentifrices on surface roughness and gloss of resin composites. J Dent.

[24] Roselino LMR, Chinelatti MA, Alandia-Román CC, Pires-de-Souza FCP (2015). Effect of brushing time and dentifrice abrasiveness on color change and surface roughness of resin composites. Braz Dent J.

[25] Wang L, Garcia FC, Amarante de Araujo P, Franco EB, Mondelli RF (2004). Wear resistance of packable resin composites after simulated toothbrushing test. J Esthet Restor Dent.

[26] Heintze SD, Forjanic M. (2005). Surface roughness of different dental materials before and after simulated toothbrushing in vitro. Oper Dent.

[27] Cho K, Rajan G, Farrar P, Prentice L, Prusty BG. (2022). Dental resin composites: a review on materials to product realizations. Compos B Eng.

[28] Ugurlu M, Husain NA, Özcan M. (2022). Repair of bulk-fill and nanohybrid resin composites: effect of surface conditioning, adhesive promoters, and long-term aging. Materials (Basel).

[29] Nguyen JF, Migonney V, Ruse ND, Sadoun M. (2012). Resin composite blocks via high-pressure high-temperature polymerization. Dent Mater.

[30] Abdalla MM, Ali IAA, Khan K (2021). The influence of surface roughening and polishing on microbial biofilm development on different ceramic materials. J Prosthodont.

[31] Samra APB, Pereira SK, Delgado LC, Phillipini Borges C. (2008). Color stability evaluation of aesthetic restorative materials. Braz Oral Res.

[32] Souza LFB, Soares PM, Ribeiro VF, Scotti N, Kleverlaan CJ, Bacchi A (2023). Influence of coloring techniques on the surface characteristics and color stability of a monolithic zirconia ceramic. J Prosthet Dent.

[33] Çakmak G, Oosterveen-Rüegsegger AL, Akay C, Schimmel M, Yilmaz B, Donmez MB. (2023). Influence of polishing technique and coffee thermal cycling on the surface roughness and color stability of additively and subtractively manufactured resins used for definitive restorations. J Prosthodont.

[34] Colak G, Katirci G. (2023). In Vitro evaluation of the effects of whitening toothpastes on the color and surface roughness of different composite resin materials. BMC Oral Health.

